# DIFFERENCES IN NEUROPSYCHIATRIC SYMPTOMS AMONG RACIAL AND ETHNIC GROUPS LIVING WITH DEMENTIA IN HOME HEALTHCARE

**DOI:** 10.1093/geroni/igac059.1827

**Published:** 2022-12-20

**Authors:** Rebecca Lassell, Kimberly Convery, Jason Fletcher, Tracy Chippendale, Shih-Yin Lin, Tessa Jones, Aditi Durga, Abraham Brody

**Affiliations:** New York University, New York, New York, United States; New York University, New York, New York, United States; New York University, New York, New York, United States; New York University, New York, New York, United States; New York University, New York, New York, United States; New York University, New York, New York, United States; New York University, New York, New York, United States; HIGN at the NYU Rory Meyers College of Nursing, New York, New York, United States

## Abstract

The relationship between dementia and neuropsychiatric symptoms is well documented. Yet, little is known about the prevalence of neuropsychiatric symptoms among diverse homebound persons living with dementia. Guided by an intersectionality framework we asked: 1) Is there an association between the presence of individual neuropsychiatric symptoms and racial and ethnic groups? 2) If so, do these symptoms differ by dementia stage among groups? We conducted a cross-sectional study of n=190 receiving skilled home healthcare in Utah, New York, and New Jersey and enrolled in the DSM-H trial. We prospectively measured symptom prevalence with the Neuropsychiatric Inventory Questionnaire and dementia stage using the Quick Dementia Rating System. We performed Chi-square tests to determine the association of individual symptom prevalence with race and ethnicity and cross tabs to descriptively stratify individual symptom prevalence by dementia stage among groups. Participants were 11.9% Hispanic, 28% non-Hispanic Black, 56.1% non-Hispanic white (white). The prevalence of delusions was significantly higher in Hispanic and non-Hispanic Black groups than whites (41.7% & 41.8% vs. 22.5%) and elation/euphoria in Hispanic and non-Hispanic Black groups than whites (20.8% &12.7% vs. 5.4%). Delusions were most prevalent in non-Hispanic Black groups with mild dementia (53.8%) and Hispanics with moderate dementia (50.1%), while elation/euphoria was most prevalent for Hispanics with severe dementia (33.3%). Other neuropsychiatric symptoms showed no significant differences. These findings expand our knowledge of differences in neuropsychiatric symptoms among racial and ethnic persons living with dementia, which can inform future studies and targeted interventions that address disparities and improve care.

